# Pulmonary Embolism in the Elderly: From Symptoms to Speckle Tracking Echocardiography

**DOI:** 10.3390/jcdd12010015

**Published:** 2024-12-31

**Authors:** Christos Ballas, Dimitrios Sfairopoulos, Ioanna Samara, Lampros Lakkas, Olga Kardakari, Athanasios Konstantinidis, Katerina K. Naka, Lampros K. Michalis, Christos S. Katsouras

**Affiliations:** 1 Department of Cardiac Surgery, University Hospital of Ioannina, 455 00 Ioannina, Greece; ballaschristos@gmail.com; 2 First Department of Cardiology, University Hospital of Ioannina, 455 00 Ioannina, Greece; dimitrios.sfairopoulos@gmail.com; 3 Service de Cardiologie, Hôpitaux du Pays du Mont Blanc, 747 00 Sallanches, France; ioan.samara31@gmail.com; 4 Department of Physiology, Faculty of Medicine, University of Ioannina, 451 10 Ioannina, Greece; ftpcavalier52@gmail.com; 5 Second Department of Cardiology, University Hospital of Ioannina, 455 00 Ioannina, Greece; olgakardakari@yahoo.gr (O.K.); drkknaka@gmail.com (K.K.N.); lamprosmihalis@uoi.gr (L.K.M.); 6 Respiratory Department, University Hospital of Ioannina, Respiratory Medicine, 455 00 Ioannina, Greece; athkon@yahoo.com; 7 Faculty of Medicine, School of Health Sciences, University of Ioannina, 451 10 Ioannina, Greece

**Keywords:** pulmonary embolism, elderly, echocardiography

## Abstract

(1) Background: There are little data about the differences in clinical and echocardiographic characteristics between elderly (aged ≥ 65 years) and younger patients with acute pulmonary embolism (PE). (2) Methods: Consecutive patients diagnosed with PE in a tertiary hospital were identified. Clinical characteristics, biomarkers and transthoracic echocardiography indices including right ventricular free wall longitudinal strain (RV-FWLS) were recorded. (3) Results: Of 200 patients enrolled, 19 patients had high-risk PE and were excluded from the study. Compared to younger patients, elderly patients with PE had less frequently pain and typical symptoms and more often were hospitalized due to another reason before the PE diagnosis. The elderly had higher values of D-dimer, high-sensitivity troponin I and brain natriuretic peptide (BNP). Echocardiographic differences were noted and the elderly had lower values of pulmonary vascular acceleration time, RV E/A ratio and lower tricuspid annular plane systolic excursion/pulmonary artery systolic pressure ratio. The RV-FWLS index did not show a statistically significant difference in distribution between age groups ≥ 65 and <65 years old. The RV diameter was similar across all age groups. (4) Conclusions: The elderly have differences compared with younger patients with PE regarding the mode of presentation, the values of biomarkers like D-dimer, BNP and troponins and some echocardiographic indices of RV affection.

## 1. Introduction

Aging is characterized by an increasing prevalence of many conditions and diseases [1,2]. Regarding thromboembolic risk, the greater the age, the higher the risk [3]. In individuals aged ≥ 80 years, the incidence of venous thromboembolism (VTE) is 2.91–12.04/1000/year, and pulmonary embolism (PE) accounts for more than one third of VTE cases [3]. Elderly PE patients have higher mortality and morbidity [4], common comorbidities (e.g., cancer, heart failure, respiratory failure and reduced mobility or immobility for a variety of reasons), less specific symptoms and a higher risk of recurrence and bleeding complications than younger patients with PE [5,6]. Besides the different mode of presentation and the higher risk of complications, aging is associated with a greater activation of the coagulation system and with more intense organ damage reflected in a more significant increase in damage-related biomarkers in patients with acute PE. The age-adjusted D-dimer cut-off value has been proposed for patients older than 50 years (higher values for older individuals) and these higher values have higher specificity and unreduced sensitivity in elderly and very elderly patients with PE [7]. Moreover, age-adjusted high sensitivity troponin levels may be more useful in the risk assessment of elderly patients with PE [8].

Published data regarding the use of transthoracic echocardiography (TTE) in elderly patients with PE are sparse. Although right ventricular dysfunction (RV_d_) can be documented and used as a useful tool for risk stratification in all-comers hemodynamically stable (non-high-risk) patients with acute PE [9], a multicenter study from Switzerland showed that the assessment of RV_d_ might not have the same value in the elderly [10]. RV_d_ was not associated with 30-day outcomes in patients aged ≥ 65 years in the setting of acute PE [10]. In addition, data from the RIETE registry showed that younger age was one of the main predictors of early TTE after PE [11]. That means that a lower percentage of elderly patients with suspected PE underwent TTE compared with younger patients.

Experimental studies have shown that aging leads to RV free wall (RVFW) fibrosis [12]. A Chinese study of healthy volunteers has proposed age-stratified normal range values for the right heart’s function using tissue tracking cardiac magnetic resonance imaging [13]. Although abnormal echocardiographic strain-related indices are common in patients with PE [14], no data exist about 2-dimensional speckle tracking echocardiography in elderly patients with PE.

We examined an unselected population with PE and investigated if there were differences in clinical, biochemical and echocardiographic characteristics (including mechanical strain indices of RV) between elderly and younger patients with non-high risk PE.

## 2. Materials and Methods

The current report is part of a prospective cohort study of consecutive non-COVID-19-related PE patients, conducted at the University Hospital of Ioannina, in Northwestern Greece. The study was approved by the Scientific Research Committee of the Hospital and complies with the Declaration of Helsinki. Informed consent was obtained from all patients entered the study.

### 2.1. Study Protocol and Patient Selection

The details of the study have been published elsewhere [14]. Briefly, the inclusion criteria were the following: (a) age ≥ 18 years and (b) documentation of thrombotic PE. For diagnosis, results from computed tomography pulmonary angiography (CTPA) or from high-probability perfusion lung scan in combination with a normal chest X-ray and clinical judgement were used. Patients were admitted to the hospital due to PE (“out-of-hospital PE”) or suffered from PE during their hospitalization for another reason in any department of the hospital (“in-hospital PE”). Patients with COVID-19-related PE [tested positive for severe acute respiratory syndrome-coronavirus-2 (SARS-CoV-2) in the previous 30 days or during their hospitalization for PE] were excluded. Patients that were hospitalized in the internal care unit during PE and the high-risk patients (these with accompanying hemodynamic instability) were also excluded (the diagnosis of PE in these patients was based on CTPA or TTE where CTPA could not be conducted for time-saving purposes).

A recording of a detailed medical history including demographics, past medical conditions and surgeries, risk factors and family history for VTE or PE-related symptoms (dyspnea, pain, hemoptysis, syncope and limb pain) was made. All the parameters included in the Geneva and Wells score and the Pulmonary Embolism Severity Index (PESI) as well as the electrocardiogram (ECG) at diagnosis were recorded. Body mass index [weight (Kg)/height (m^2^)] and estimated glomerular filtration rate [eGFR (MDRD calculation)] were calculated [15].

### 2.2. Biochemical Markers

High sensitivity cardiac Troponin-I (hs-cTnI, Beckman Coulter, Inc., Brea, CA, USA) and D-dimer (STA-Liatest D-Di PLUS kit using STA R Max3 and STA Compact Max3 analyzers, Diagnostica Stago S.A.S., Asnières sur Seine, France) levels were measured during the diagnostic work-up one or more times and the reported values (pg/mL for troponin and fibrinogen equivalent units per mL, FEU/mL for D-Dimers) were the “closest” to the diagnosis, in time. Brain natriuretic peptide (BNP) measurement (Quidel Triage BNP test for Beckman Coulter Access Family of Immunoassay Systems using ethylene diamine tetraacetic acid as the anticoagulant, pg/mL) was measured in all cases (in most cases, immediately after the diagnosis of PE). The assay’s 99th centile for cTnI is 11.6 pg/mL for women (95% CI 8.4–18.3) and 19.8 pg/mL for men (95% CI 14.0–42.9). The expected normal level for D-dimers is <0.5 μg/mL and for BNP ≤ 100 pg/mL (in patients without heart failure).

### 2.3. Echocardiography

Bedside TTE was performed at the time of diagnosis and in any case not later than 24 h from that time point. The echocardiographic signs mentioned in the European Guidelines for the diagnosis and treatment of PE as markers of RV dysfunction or pressure overload (RVd/po) were considered as “conventional” markers of RV_d/po_ [16]. These signs include the following: abnormal enlargement of RV or RV/left ventricle basal diameter greater than >1, McConnell sign and dyskinesia or hypokinesia of the free right ventricular wall, abnormal movement of the intraventricular septum, “60/60 sign”, tricuspid annular plane systolic excursion (TAPSE) < 16 mm and peak systolic velocity (S′) of tricuspid annulus < 9.5 cm/s [16,17,18]. Right ventricular free wall longitudinal strain (RV-FWLS) was calculated offline using the Tom Tec 2D Cardiac Performance Image Analysis (Unterschleissheim, Germany) and values > −20% were considered as abnormal [17,18]. Fractional area change (FAC) index (the difference between the RV end-diastolic and end-systolic measured from an apical four-chamber view) [18], the ratio TAPSE/pulmonary artery systolic pressure (TAPSE/PASP) [19], mean pulmonary arterial pressure (mPAP) and pulmonary artery resistance (PVR) were measured when it was feasible. The Doppler evaluation of PVR was performed using the following equation: PVR in wood units (WU) = 10 × peak tricuspid regurgitant velocity (TRVmax in m/s)/right ventricular outflow tract (RVOT) time-velocity integral (TVI_RVOT_) (cm) + 0.16 [20]. Mean PAP calculated from the peak velocity of the pulmonary regurgitation jet Doppler signal (PRpeak velocity) was obtained using the following formula: mPAP = 4 × (PRpeak velocity)^2^ + right atrial pressure (RAP) [21].

### 2.4. Statistical Analysis

In our analysis, elderly patients were considered as those with an age of ≥65 years. Continuous variables were presented as mean (standard deviation) or median [interquartile range], based on their distribution. Normality was assessed using the Kolmogorov–Smirnov or Shapiro–Wilk test, as appropriate. Spearman’s rank correlation coefficient (Spearman’s rho, ρ) was used to assess the strength and direction of the association between age, RV-FWLS, echocardiographic parameters and echocardiography-derived hemodynamics. For normally distributed data, comparisons were made with an independent samples *t*-test if variance homogeneity was confirmed by Levene’s test; otherwise, Welch’s *t*-test was used. For non-normally distributed data, the Mann–Whitney U test was applied. Categorical variables were presented as percentages and compared using the chi-square test or Fisher’s exact test, as appropriate. A 2-sided *p*-value of <0.05 indicated statistical significance. All analyses were conducted using SPSS V.29 (IBM Corp., New York, NY, USA).

## 3. Results

From September 2019 to June 2022, a total of 181 (82 women) consecutive patients with documented, non-high risk, non-COVID-related PE were included in the study. The baseline demographics and clinical characteristics of these patients are presented in Table 1. The median age of the study cohort was 72 [59, 79] years, while 54.7% of patients were male. Among the cohort, 63.5% had hypertension, 27.1% had a history of cancer, 22.1% had diabetes mellitus and 1.7% had a history of thrombophilia. None of the patients were pregnant and 12.7% had a history of VTE. Additionally, 51.9% of the patients were admitted to the hospital from the emergency department (the remaining were already hospitalized for another reason). The median eGFR was 72 (57, 88) mL/min/1.73 m^2^. About half of patients (51.4%) had abnormal ECG (sinus tachycardia, atrial arrhythmias or signs of RV affection). Differences were noted regarding baseline demographics and clinical characteristics (Table 1).

Elderly patients had a higher prevalence of hypertension (*p* < 0.001), worse renal function (as indicated by eGFR, *p* < 0.001), a higher prevalence of diabetes mellitus (*p* = 0.014), a lower rate of thrombophilia (*p* = 0.014) and they were less likely to be former or active smokers, but especially active smokers (*p* < 0.001). The proportion of patients who had risk factors for VTE and patients already on anticoagulation therapy did not differ in all groups, while elderly patients were more often hospitalized for another reason in the hospital (*p* = 0.021).

Regarding the mode of presentation, elderly patients presented less frequently with chest pain (*p* = 0.004) and with a combination of symptoms (*p* = 0.022), and more frequently with non-typical symptoms (*p* = 0.033). Forty-two (23.2%) patients were receiving treatment with beta blockers, 42 (23.2%) were on anticoagulation therapy and 16 (8.8%) presented with atrial fibrillation or flutter (Table 2).

**Figure 1:**
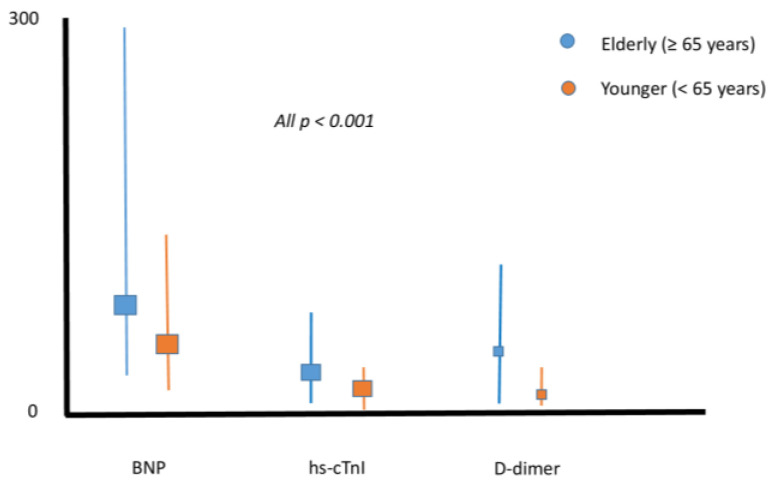
BNP (pg/mL), hs-cTnI (pg/mL) and D-dimer (μg/dL, for uniformity reasons) in younger and elderly patients. BNP: brain natriuretic peptide; hs-cTnI: high-sensitivity cardiac troponin I.

The levels of hs-TnI, D-Dimers and BNP (Figure 1) were also significantly higher in the older age groups across both age comparisons (*p* < 0.001 for all).

**Figure 2:**
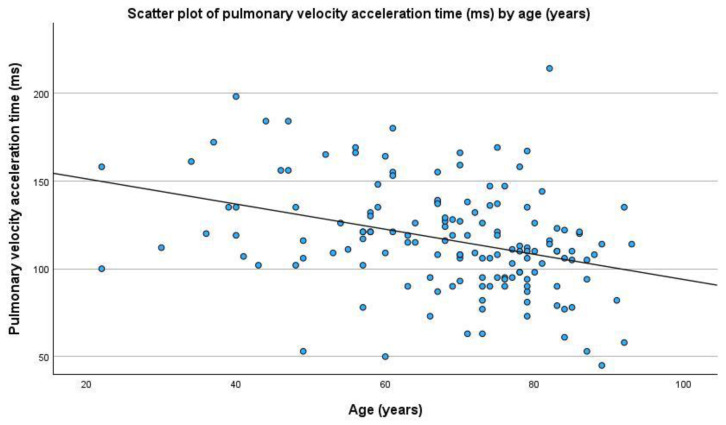
Correlation between age and pulmonary velocity acceleration time in milliseconds (ms) (ρ = −0.365, *p* < 0.001).

In relation to the echocardiographic parameters, the proportion of patients with conventional indices of RV pressure or volume overload was 23% and there were no significant differences across age groups ≥ 65 vs. <65 for any particular conventional index of RV_d/PO_. In 34 patients, no a detailed echocardiographic study focusing on RV-FWLS was recorded, mainly due to a very poor image quality and/or echocardiographic window. Age ranged from 22 to 95 years (mean age, 68.52 years; standard deviation, 14.66 years). RV-FWLS values ranged from −36.90% to −4.00% with a mean value of −18.98% and a standard deviation of 4.89%. Age was correlated with RV-FWLS (ρ = 0.188, *p* = 0.022, indicating a decrease in its absolute value with increasing age), right atrial area, (ρ = 0.203, *p* = 0.011), PASP (ρ = 0.189, *p* = 0.020), pulmonary velocity acceleration time (ρ = −0.365, *p* < 0.001; Figure 2), FAC (ρ = −0.168, *p* = 0.041), mean PAP (ρ = 0.480, *p* = 0.003), PVR (ρ = 0.176, *p* = 0.045), TAPSE (ρ = −0.175, *p* = 0.030) and TAPSE/PASP ratio (ρ = −0.274, *p* = 0.001). The rest of the examined variables did not show statistically significant correlations with age.

RV-FWLS did not show a statistically significant difference in distribution between the age groups ≤ 65 and >65, with values of −19.75 [−21.93, −17.35] in the ≤ 65 group and −19 [−21.6, −16.35] in the >65 group (*p* = 0.184) (Table 3).

**Figure 3:**
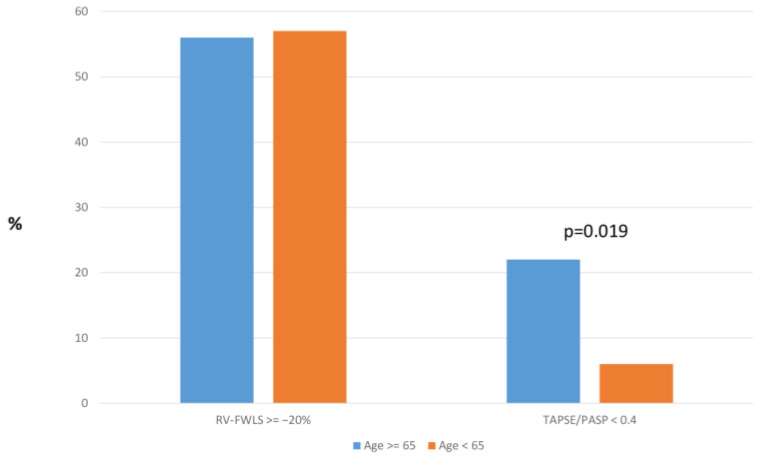
Proportion of patients (≥65 and <65 years) with RV-FWLS ≥ −20% TAPSE/PASP < 0.4. RV-FWLS: right ventricular free wall longitudinal strain; TAPSE: tricuspid annular plane systolic excursion; PASP: pulmonary artery systolic pressure.

Regarding the proportion of patients with RV-FWLS > −20%, the ≥65 and <65 groups had similar percentages (56% and 57%, respectively; *p* = 0.935) (Figure 3).

Pulmonary velocity acceleration time distributions differed significantly, with values of 121 [111, 156] in the <65 group and 108.5 [93.75, 126] in the ≥65 group (*p* < 0.001). The RV E/A ratio showed a significantly different distribution in the ≥65 versus <65 comparison, with values of 1.1 [0.82, 1.3] in the < 65 group and 0.93 [0.75, 1.1] in the ≥ 65 group (*p* = 0.020), while the ratio of TAPSE/PASP was lower in elderly patients (*p* = 0.031). For the proportion of patients with a TAPSE/PASP ratio < 0.4, a significant difference was observed across both age comparisons. In the <65 vs. ≥65 groups, 6.3% of patients aged ≤ 65 had values below this threshold, compared to 21.7% of those aged ≥ 65 (*p* = 0.019).

## 4. Discussion

In this observational study, we investigated the differences in clinical, biochemical and primarily echocardiographic characteristics between elderly and younger patients with non-high risk, non-COVID-19-related PE. The main finding of this study was that the elderly had differences compared with younger patients with PE regarding the mode of presentation, the values of biomarkers like D-dimer, BNP and troponins and some echocardiographic indices of RV_d/po_. Differences were noted regarding RV E/A ratio, pulmonary artery acceleration time distribution and TAPSE/PASP ratio. The RV-FWLS index did not show a statistically significant difference in distribution between age groups ≤ 65 and >65.

The elderly population is at an increased risk for PE compared to younger patients. Punucollu et al. reported that the elderly may have an atypical presentation of PE. Syncope happened in about one in five elderly (≥65 years) patients with PE and was more common in this age group compared with younger patients. Furthermore, cancer was the most common risk factor in the above-mentioned patient’s group of age [22]. In our study, syncope was not a common symptom, and the proportion of the patients with syncope or cancer did not differ between age groups. The proportion of patients with out-of-hospital PE was smaller in patients aged ≥ 65 years. The latter may explain that pain was present in fewer elderly patients (possibly due to taking analgesics during hospitalization for other reasons).

In the current study, the median BNP value was 2.5 times higher in the elderly population. Previous studies have shown that BNP levels may be 3 times higher in the elderly population without PE, especially in patients with renal dysfunction and left ventricular hypertrophy [23]. In our elderly population, eGFR was lower and hypertension was a more frequent comorbidity. Similarly, the elderly had higher hs-cTnI and D-dimer levels. The median hs-cTnI level was above the 99th percentile, and the median D-dimer levels were 10 times higher than the cut-off value of 0.5 μg/mL [24]. Regarding troponin, we have previously shown that myocardial injury in elderly patients with severe renal impairment is a malignant condition [25].

Studies about the role of echocardiography in the setting of PE in elderly populations have shown conflicting results. Some investigators have reported that RV dysfunction in elderly patients with PE (i.e., RV dilation, RV end-systolic area, RV hypokinesia) is correlated with the extent of PE [26]. Osken et al. showed that markers indicating RV dysfunction, such as RV dilatation, TAPSE < 16 mm and S velocity < 10 cm/s, were more common in the older population with PE, while increased RV end-diastolic basal diameter and TAPSE < 16 mm were associated with increased mortality in this population [27]. In contrast to these studies, other evidence suggests that there is no association between RV dysfunction and clinical outcomes in elderly patients with PE [10,28]. On the other hand, studies focusing on younger populations with non-high-risk PE have found that RV dysfunction has prognostic value for risk stratification and short-term mortality, especially when combined with myocardial injury markers [29,30]. In our study, pulmonary vascular acceleration time, RV E/A and TAPSE/PASP ratio were differed in elderly patients. Strain indices can assess myocardial deformation and detect subclinical myocardial damage at early stages, proven valuable for both left and right ventricular diseases [31,32]. Recently, our group demonstrated that RV-FWLS is the most abnormal echocardiographic marker in patients with PE [14]. Trivedi et al., in their retrospective study of 84 patients with PE and 66 healthy controls, showed that combining RV size and function measurements with RV-FWLS also had an important discriminative power (AUC 0.921, SE 0.024) for PE patients compared to models that did not include RV-FWLS [33]. Ramberg et al. demonstrated that the basal and mid segments of the free wall are more affected in acute PE, suggesting that their evaluation is essential in the emergency departments [34]. Alongside the impaired free wall longitudinal strain, septal segments may also be affected in PE [35,36]. Wilinski et al. noted an alteration specifically in the mid-segment of the free wall, with better reliability and reproductivity, which could be explained by the more pronounced mechanical movement of the basal part of the RVFW [37]. The midventricular RV systolic strain rate can also be used to diagnose acute cor pulmonale with 83.3% sensitivity and 78.6% specificity at a cutoff of −12.2%, while it can enable the differentiation between acute and chronic forms of the disease [38].

Longitudinal strain of the RV may contribute to prognostication in PE patients. Dahhan et al. demonstrated an association between RVFWS, the RV Tei index and 30-day mortality when added to clinical parameters [39]. Similarly, Lee et al. showed that RV-FWLS was the best prognostic imaging index for the prediction of in-hospital mortality in patients with acute non-high-risk PE in a multivariate analysis after the adjustment of confounding factors (OR 1.20, 95% CI 1.07–1.35, *p* = 0.002) [40]. According to the study of Batur G. K et al., the patients with acute PE who died during follow-up at one year after the episode had a lower RV-FWLS in diagnosis than those who survived [13.6 ± 3.6 (−%) and 18.4 ± 4.6 (−%), respectively, *p* < 0.001] [41]. In the current study, RV-FWLS values did not differ between the two groups. Moreover, the elderly group had the same proportion of patients with ≥−20% RV-FWLS compared with the younger group. However, there was not a group of patients with suspected PE but another final diagnosis to examine the specificity and sensitivity of the index. Studies with a larger population would add information about other echocardiographic indices or a combination of indices (e.g., RV-FWLS and pulmonary artery acceleration time or TAPSE/PASP) in elderly patients with PE. The use of novel markers may be especially beneficial for elderly patients, in whom the presence of comorbidities can complicate the diagnosis, resulting in a delay in therapy and worse outcomes [42].

### Limitations

The study has some limitations. Firstly, this was an observational single center study, and the findings may not be generalizable to broader populations. Secondly, the number of patients was relatively small. Thirdly, unmeasured variables may have influenced the outcomes. Fourthly, no causal inferences can be made given the study design. Lastly, no control group (patients with suspected PE and an other final diagnosis) existed.

## 5. Conclusions

In summary, elderly patients with PE differ in the mode of presentation compared to younger patients and they have higher levels of troponin, D-dimer and BNP. The elderly have a lower pulmonary vascular acceleration time, RV E/A ratio and TAPSE/PASP ratio. Further studies are needed in order to clarify if these differences could be a valuable tool in detecting, stratifying and predicting the echocardiographic deterioration of RV or clinical outcomes in elderly patients with non-high-risk PE.

## Figures and Tables

**Table 1:** Baseline characteristics of the study patients.

Variable	Age < 65 Years (*n* = 61)	Age ≥ 65 Years (*n* = 120)	*p* Value
Age, years	54 [46, 59.5]	77 [72, 82]	<0.001
Men	34 (55.7%)	65 (54.2%)	0.841
BMI, kg/m^2^	27.8 [24.9, 32.0]	27.7 [24.5, 31.2]	0.553
Diabetes mellitus	7 (11.5%)	33 (27.5%)	0.014
Hypertension	25 (41.0%)	90 (75.0%)	<0.001
eGFR, mL/min/1.73 m^2^	85.1 [71.7, 99.56]	64 [50.5, 80.96]	<0.001
Thrombophilia	3 (4.9%)	0 (0%)	0.037
Pregnancy	0 (0%)	0 (0%)	N/A
Active cancer	14 (23.0%)	35 (29.2%)	0.374
Out of hospital PE	39 (63.9%)	55 (45.8%)	0.021
Active smoker	26 (42.6%)	22 (18.3%)	<0.001
Anticoagulant therapy	13 (21.3%)	29 (24.2%)	0.667
Strong risk factors *	24 (39.3%)	42 (35.0%)	0.566
Moderate risk factors *	24 (39.3%)	56 (46.7%)	0.348
Weak risk factors *	13 (21.3%)	22 (18.3%)	0.632

Values are presented as *n* (%), mean (SD) or median [IQR]. BMI: body mass index; eGFR: estimated glomerular filtration rate; PE: pulmonary embolism. * Based on the classification of the European Guidelines for the diagnosis and treatment of pulmonary embolism [16].

**Table 2:** Clinical characteristics, biomarkers and atrial arrhythmias of the study patients.

Variable	Age ≥ 65 *(n* = 61*)*	Age < 65 *(n* = 120*)*	*p* Value
Symptoms			
No symptoms	4 (6.6%)	13 (10.8%)	0.351
Dyspnea	37 (60.7%)	69 (57.5%)	0.684
Chest pain	27 (44.3%)	28 (23.3%)	0.004
Lower limb pain	7 (11.5%)	6 (5%)	0.132
Syncope	2 (3.3%)	6 (5%)	0.719
Hemoptysis	5 (8.2%)	5 (4.2%)	0.308
Cough	4 (6.6%)	15 (12.5%)	0.218
Non-typical symptoms	4 (6.6%)	22 (18.3%)	0.033
Combination of symptoms	26 (42.6%)	31 (25.8%)	0.022
Biomarkers			
hs-Tn I (pg/mL)	6.4 [3.5, 26.4]	23.5 [8.63, 94.68]	<0.001
D-Dimers (µg/mL)	2.92 [1.19, 5.94]	4.49 [2.43, 10.34]	<0.001
BNP (pg/mL)	46 [22.5, 123.5]	112 [58, 298]	<0.001
Atrial arrhythmias			
Atrial fibrillation or atrial flutter	2 (3.3%)	14 (11.7%)	0.06
Paroxysmal atrial fibrillation	2 (3.3%)	7 (5.8%)	0.720

Values are presented as *n* (%), mean (SD) or median [IQR]. hs-TnI: high-sensitivity troponin I; BNP: brain natriuretic peptide.

**Table 3:** Echocardiographic parameters and echocardiography-derived hemodynamics between age groups ≥ 65 and <65 in patients with non-high risk PE.

Variable	Age < 65 *(n)*	Age ≥ 65 *(n)*	Age < 65	Age ≥ 65	*p* Value
RV-FWLS, %	50	97	−19.75 [−21.93, 17.35]	−19.00 [−21.6, −16.35]	0.184
RA area, cm^2^	54	102	13.9 [11.5, 15.98]	14.35 [11.8, 18.7]	0.132
PASP, mmHg	49	102	31 [21, 39]	33 [23, 47]	0.136
PVAT, ms	51	98	121 [111, 156]	109 [94, 126]	<0.001
IVCT TDI, s	48	87	0.12 [0.10, 0.15]	0.11 [0.09, 0.14]	0.192
RV E/A	41	68	1.10 [0.8, 1.3]	0.93 [0.8, 1.1]	0.020
RV E/e’	34	65	5.12 [4.01, 6.29]	5.63 [3.58, 6.95]	0.359
FAC, %	50	99	46 [37, 50]	42 [37, 48]	0.197
RV 4-ch mid-dimension, cm	53	103	2.92 (0.50)	2.91 (0.63)	0.959
RV 4-ch long-dimension, cm	53	102	7.08 (0.76)	7.03 (0.83)	0.719
mean PAP, mmHg	7	30	12 [7, 21]	19 [14, 34]	0.092
PVR, WU	44	87	1.59 [1.14, 2.07]	1.64 [1.3, 2.16]	0.233
Isovolumic acceleration, cm/s^2^	44	83	40 [33, 56]	40 [33, 54]	0.694
TAPSE, cm	53	101	2.21 (0.40)	2.10 (0.46)	0.157
TAPSE/PASP, mm/mmHg	48	92	0.80 [0.58, 0.95]	0.61 [0.42, 0.95]	0.031

Values are presented as mean (SD) or median [IQR]. RV: right ventricle; FWLS: free wall longitudinal strain; RA: right atria; PASP: pulmonary artery systolic pressure; PVAT: pulmonary artery acceleration time; IVCT: isovolumic contraction time; TDI: tissue doppler imaging; FAC: fractional area change; ch: chamber; PAP: pulmonary artery pressure; PVR: pulmonary artery resistance; WU: Wood units; TAPSE: tricuspid annular plane systolic excursion.

## Data Availability

Data will be available upon request.

## References

[1] Lemoine M. (2020). Defining aging. Biol. Philos..

[2] Harman D. (1991). The aging process: Major risk factor for disease and death. Proc. Natl. Acad. Sci. USA.

[3] Raskob G.E., Angchaisuksiri P., Blanco A.N., Buller H., Gallus A., Hunt B.J., Hylek E.M., Kakkar A., Konstantinides S.V., McCumber M. (2014). Thrombosis: A major contributor to global disease burden. Arter. Thromb. Vasc. Biol..

[4] Tritschler T., Aujesky D. (2017). Venous thromboembolism in the elderly: A narrative review. Thromb. Res..

[5] Robert-Ebadi H., Righini M. (2014). Diagnosis and management of pulmonary embolism in the elderly. Eur. J. Intern. Med..

[6] Spencer F.A., Gore J.M., Lessard D., Emery C., Pacifico L., Reed G., Gurwitz J.H., Goldberg R.J. (2008). Venous thromboembolism in the elderly: A community-based perspective. Thromb. Haemost..

[7] Polo Friz H., Pasciuti L., Meloni D.F., Crippa M., Villa G., Molteni M., Primitz L., Del Sorbo D., Delgrossi G., Cimminiello C. (2014). A higher d-dimer threshold safely rules-out pulmonary embolism in very elderly emergency department patients. Thromb Res..

[8] Kaeberich A., Seeber V., Jiménez D., Kostrubiec M., Dellas C., Hasenfuß G., Giannitsis E., Pruszczyk P., Konstantinides S., Lankeit M. (2015). Age-adjusted high-sensitivity troponin T cut-off value for risk stratification of pulmonary embolism. Eur. Respir. J..

[9] Cimini L.A., Candeloro M., Pływaczewska M., Maraziti G., Di Nisio M., Pruszczyk P., Agnelli G., Becattini C. (2022). Prognostic role of different findings at echocardiography in acute pulmonary embolism: A critical review and meta-analysis. ERJ Open Res..

[10] Hofmann E., Limacher A., Méan M., Kucher N., Righini M., Frauchiger B., Beer J.H., Osterwalder J., Aschwanden M., Matter C.M. (2016). Echocardiography does not predict mortality in hemodynamically stable elderly patients with acute pulmonary embolism. Thromb. Res..

[11] Bikdeli B., Lobo J.L., Jiménez D., Green P., Fernández-Capitán C., Bura-Riviere A., Otero R., DiTullio M.R., Galindo S., Ellis M. (2018). Early Use of Echocardiography in Patients with Acute Pulmonary Embolism: Findings from the RIETE Registry. J. Am. Heart Assoc..

[12] Sharifi Kia D., Shen Y., Bachman T.N., Goncharova E.A., Kim K., Simon M.A. (2022). The Effects of Healthy Aging on Right Ventricular Structure and Biomechanical Properties: A Pilot Study. Front. Med..

[13] Liu T., Wang C., Li S., Zhao Y., Li P. (2019). Age- and gender-related normal references of right ventricular strain values by tissue tracking cardiac magnetic resonance: Results from a Chinese population. Quant. Imaging Med. Surg..

[14] Ballas C., Lakkas L., Kardakari O., Konstantinidis A., Exarchos K., Tsiara S., Kostikas K., Naka K.Κ., Michalis L.K., Katsouras C.S. (2023). What is the real incidence of right ventricular affection in patients with acute pulmonary embolism?. Acta Cardiol..

[15] Levey A.S., Stevens L.A., Schmid C.H., Zhang Y.L., Castro A.F., Feldman H.I., Kusek J.W., Eggers P., Van Lente F., Greene T. (2009). A new equation to estimate glomerular filtration rate. Ann. Intern. Med..

[16] Konstantinides S.V., Meyer G., Becattini C., Bueno H., Geersing G.J., Harjola V.P., Huisman M.V., Humbert M., Jennings C.S., Jiménez D. (2020). ESC Scientific Document Group. 2019 ESC Guidelines for the diagnosis and management of acute pulmonary embolism developed in collaboration with the European Respiratory Society (ERS). Eur. Heart J..

[17] Lang R.M., Badano L.P., Mor-Avi V., Afilalo J., Armstrong A., Ernande L., Flachskampf F.A., Foster E., Goldstein S.A., Kuznetsova T. (2015). Recommendations for cardiac chamber quantification by echocardiography in adults: An update from the American Society of Echocardiography the European Association of Cardiovascular Imaging. J. Am. Soc. Echocardiogr..

[18] Dutta T., Aronow W.S. (2017). Echocardiographic evaluation of the right ventricle: Clinical implications. Clin. Cardiol..

[19] Lyhne M.D., Kabrhel C., Giordano N., Andersen A., Nielsen-Kudsk J.E., Zheng H., Dudzinski D.M. (2021). The echocardiographic ratio tricuspid annular plane systolic excursion/pulmonary arterial systolic pressure predicts short-term adverse outcomes in acute pulmonary embolism. Eur. Heart J. Cardiovasc. Imaging.

[20] Abbas A.E., Fortuin F.D., Schiller N.B., Appleton C.P., Moreno C.A., Lester S.J. (2003). A simple method for noninvasive estimation of pulmonary vascular resistance. J. Am. Coll Cardiol..

[21] Parasuraman S., Walker S., Loudon B.L., Gollop N.D., Wilson A.M., Lowery C., Frenneaux M.P. (2016). Assessment of pulmonary artery pressure by echocardiography—A comprehensive review. Int. J. Cardiol. Heart Vasc..

[22] Punukollu H., Khan I.A., Punukollu G., Gowda R.M., Mendoza C., Sacchi T.J. (2005). Acute pulmonary embolism in elderly: Clinical characteristics and outcome. Int. J. Cardiol..

[23] Sayama H., Nakamura Y., Saito N., Kinoshita M. (1999). Why is the concentration of plasma brain natriuretic peptide in elderly inpatients greater than normal?. Coron. Artery Dis..

[24] Douma R.A., le Gal G., Söhne M., Righini M., Kamphuisen P.W., Perrier A., Kruip M.J., Bounameaux H., Büller H.R., Roy P.M. (2010). Potential of an age adjusted D-dimer cut-off value to improve the exclusion of pulmonary embolism in older patients: A retrospective analysis of three large cohorts. BMJ.

[25] Samara I., Tsiara S., Papafaklis M.I., Pappas K., Kolios G., Vryzas N., Michalis L.K., Bairaktari E.T., Katsouras C.S. (2021). Elderly patients with non-cardiac admissions and elevated high-sensitivity troponin: The prognostic value of renal function. World J. Cardiol..

[26] Chung T., Emmett L., Khoury V., Lau G.T., Elsik M., Foo F., Allman K.C., Kritharides L. (2006). Atrial and ventricular echocardiographic correlates of the extent of pulmonary embolism in the elderly. J. Am. Soc. Echocardiogr..

[27] Ösken A., Yelgeç N.S., Şekerci S.S., Asarcıklı L.D., Dayı Ş.Ü., Çam N. (2021). Differences in clinical and echocardiographic variables and mortality predictors among older patients with pulmonary embolism. Aging Clin. Exp. Res..

[28] Parenti N., Bonarelli S., Fanciulli A. (2005). Pulmonary embolism in younger and older adults: Clinical presentation and comparison of right ventricular dysfunction, a new prognostic echocardiographic index. Crit. Care.

[29] Becattini C., Casazza F., Forgione C., Porro F., Fadin B.M., Stucchi A., Lignani A., Conte L., Imperadore F., Bongarzoni A. (2013). Acute pulmonary embolism: External validation of an integrated risk stratification model. Chest.

[30] Pruszczyk P., Goliszek S., Lichodziejewska B., Kostrubiec M., Ciurzyński M., Kurnicka K., Dzikowska-Diduch O., Palczewski P., Wyzgal A. (2014). Prognostic value of echocardiography in normotensive patients with acute pulmonary embolism. JACC Cardiovasc. Imaging.

[31] Hamada-Harimura Y., Seo Y., Ishizu T., Nishi I., Machino-Ohtsuka T., Yamamoto M., Sugano A., Sato K., Sai S., Obara K. (2018). Incremental Prognostic Value of Right Ventricular Strain in Patients with Acute Decompensated Heart Failure. Circ. Cardiovasc. Imaging.

[32] Tadic M., Nita N., Schneider L., Kersten J., Buckert D., Gonska B., Scharnbeck D., Reichart C., Belyavskiy E., Cuspidi C. (2021). The Predictive Value of Right Ventricular Longitudinal Strain in Pulmonary Hypertension, Heart Failure, and Valvular Diseases. Front. Cardiovasc. Med..

[33] Trivedi S.J., Terluk A.D., Kritharides L., Chow V., Chia E.-M., Byth K., Mussap C.J., Ng A.C.C., Thomas L. (2020). Right ventricular speckle tracking strain echocardiography in patients with acute pulmonary embolism. Int. J. Cardiovasc. Imaging.

[34] Ramberg E., Olausson M., Jørgensen T.B.S., Nepper M.L., Bhardwaj P., Binko T.S., Petersen J.R., Fornitz G.G. (2017). Right atrial and ventricular function evaluated with speckle tracking in patients with acute pulmonary embolism. Am. J. Emerg. Med..

[35] Platz E., Hassanein A.H., Shah A., Goldhaber S.Z., Solomon S.D. (2012). Regional right ventricular strain pattern in patients with acute pulmonary embolism. Echocardiography.

[36] Sugiura E., Dohi K., Onishi K., Takamura T., Tsuji A., Ota S., Yamada N., Nakamura M., Nobori T., Ito M. (2009). Reversible right ventricular regional non-uniformity quantified by speckle-tracking strain imaging in patients with acute pulmonary thromboembolism. J. Am. Soc. Echocardiogr..

[37] Wiliński J., Skwarek A., Borek R., Medygrał M., Chrzan I., Lechowicz-Wilińska M., Chukwu O. (2023). Indexing of Speckle Tracking Longitudinal Strain of Right Ventricle to Body Surface Area Does Not Improve Its Efficiency in Diagnosis and Mortality Risk Stratification in Patients with Acute Pulmonary Embolism. Healthcare.

[38] Park J.-H., Park Y.S., Kim Y.J., Lee I.S., Kim J.H., Lee J.-H., Choi S.W., Jeong J.-O., Seong I.-W. (2011). Differentiation between acute and chronic cor pulmonales with midventricular systolic strain of the right ventricle in the emergency department. Heart Vessel..

[39] Dahhan T., Siddiqui I., Tapson V.F., Velazquez E.J., Sun S., Davenport C.A., Samad Z., Rajagopal S. (2016). Clinical and echocardiographic predictors of mortality in acute pulmonary embolism. Cardiovasc. Ultrasound.

[40] Lee K., Kwon O., Lee E.-J., Sin M.-J., Lee J.S., Lee S., Kang D.-H., Song J.-K., Song J.-M. (2019). Prognostic value of echocardiographic parameters for right ventricular function in patients with acute non-massive pulmonary embolism. Heart Vessel..

[41] Kanar B.G., Göl G., Oğur E., Kavas M., Ataş H., Mutlu B. (2019). Assessment of right ventricular function and relation to mortality after acute pulmonary embolism: A speckle tracking echocardiography-based study. Echocardiography.

[42] Masotti L., Ray P., Righini M., Le Gal G., Antonelli F., Landini G., Cappelli R., Prisco D., Rottoli P. (2008). Pulmonary embolism in the elderly: A review on clinical, instrumental and laboratory presentation. Vasc. Health Risk Manag..

